# Glycolysis under Circadian Control

**DOI:** 10.3390/ijms222413666

**Published:** 2021-12-20

**Authors:** Jana Zlacká, Michal Zeman

**Affiliations:** Department of Animal Physiology and Ethology, Faculty of Natural Sciences, Comenius University in Bratislava, Ilkovicova 6, 842 15 Bratislava, Slovakia; horvathov201@uniba.sk

**Keywords:** glycolysis, metabolic reprogramming, oxidative phosphorylation, circadian, clock genes

## Abstract

Glycolysis is considered a main metabolic pathway in highly proliferative cells, including endothelial, epithelial, immune, and cancer cells. Although oxidative phosphorylation (OXPHOS) is more efficient in ATP production per mole of glucose, proliferative cells rely predominantly on aerobic glycolysis, which generates ATP faster compared to OXPHOS and provides anabolic substrates to support cell proliferation and migration. Cellular metabolism, including glucose metabolism, is under strong circadian control. Circadian clocks control a wide array of metabolic processes, including glycolysis, which exhibits a distinct circadian pattern. In this review, we discuss circadian regulations during metabolic reprogramming and key steps of glycolysis in activated, highly proliferative cells. We suggest that the inhibition of metabolic reprogramming in the circadian manner can provide some advantages in the inhibition of oxidative glycolysis and a chronopharmacological approach is a promising way to treat diseases associated with up-regulated glycolysis.

## 1. Introduction

Most cells produce the majority of adenosine triphosphate (ATP) via the metabolic pathway known as oxidative phosphorylation (OXPHOS), which uses energy released by nutrient oxidation. OXPHOS is an enzymatic process defined as an electron transport-linked reaction coupled to the ATP synthesis through an electrochemical transmembrane gradient. This process requires a sufficient amount of oxygen [1]. Under a condition of oxygen deprivation, some of the cells are able to switch from OXPHOS to the glycolytic ATP production. An up-regulated rate of glycolysis and abundant production of glycolysis intermediates are needed for the biosynthesis of macromolecules in the pentose phosphate pathway and serine biosynthesis pathway [2]. 

Glycolysis is a 10-step metabolic pathway, which results in the production of pyruvate and two molecules of ATP. Each step is catalysed by a specific enzyme or a group of enzymes. Briefly, upon entering the cell, glucose is phosphorylated to glucose-6-phosphate (G6P) by the enzyme hexokinase 2 (HK2) in the first and rate-limiting reaction of glycolysis. Glucose-6-phosphate can enter the glycolytic or pentose phosphate pathway. Next, the enzyme glucose-6-phosphate isomerase catalyses the conversion of G6P to fructose-6-phosphate (F6P), which is converted to fructose-1,6-bisphosphate by the enzyme phosphofructokinase-1 (PFK-1). This enzyme uses ATP as the energy source. In the last step of glycolysis, ATP and pyruvate are produced by the enzyme pyruvate kinase [3], which catalyses the irreversible transfer of the phosphoryl group from phosphoenolpyruvate to ADP. Overall, the net products of glycolysis are two molecules of ATP, two molecules of nicotinamide adenine dinucleotide (NADH), and pyruvate [3]. 

Glycolysis is regulated by specific enzymes; among them, 6-phosphofructo-2-kinase/fructose-2,6-bisphosphatase-3 (PFKFB3) is the most significant regulator. The PFKFB3 enzyme contributes to the synthesis of fructose-2,6-bisphosphate (F2,6P2), an allosteric activator of PFK-1 [4]. The concentration of F2,6P2 is controlled by a family of bifunctional PFKFB enzymes encoded by four genes (*pfkfb 1–4*). Of all PFKFB isoenzymes, the PFKFB3 has a 700-fold higher kinase than bisphosphatase activity, contributing to the production of F2,6P2 [5]. Moreover, enzyme PFKFB3 is the most abundant isoenzyme in endothelial cells and in several tumour cell lines after exposure to growth factors. 

In general, healthy cells rely on OXPHOS and glycolysis for ATP production; however, the energy contribution differs depending on the cell type and cellular microenvironment. Under a normal condition of sufficient oxygen, 70% of ATP is produced in OXPHOS. Conversely, in a hypoxic condition, the OXPHOS is weakened; therefore, the process of glycolysis is enhanced. The balance between OXPHOS and glycolysis helps to maintain cellular energy homeostasis [1]. 

Aerobic glycolysis is considered a dominant metabolic pathway in activated endothelial, immune, and cancer cells. Most of the glucose is also converted to lactate when oxygen is available (Warburg effect). During the aerobic form of glycolysis, approximately four molecules of ATP are produced per glucose molecule [3]. Despite the fact that ATP production is significantly lower compared to OXPHOS, these cells prefer aerobic glycolysis when glucose is not the limiting factor. If oxygen is available, enzyme pyruvate dehydrogenase (PDH) converts pyruvate to acetyl-Co-A, which enters the Krebs cycle in the mitochondria. Acetyl-Co-A is then oxidized to carbon dioxide in cellular respiration. During oxidative phosphorylation, 36 molecules of ATP are produced per glucose molecule. In the absence of oxygen, pyruvate is converted to lactate in a process known as anaerobic glycolysis. This conversion is catalysed by lactate dehydrogenase (LDH) coupled with NADH to NAD+ oxidation. In anaerobic glycolysis, two molecules of ATP are produced from one molecule of glucose [1]. 

This review provides an overview of current knowledge about glycolysis in highly proliferative cells, including endothelial, epithelial, immune, and cancer cells. Glucose metabolism is under strong circadian control; therefore, we discuss the physiology of the circadian system. Moreover, we focus on a detailed description of factors regulating glycolysis in a circadian manner and identify the links between the glucose metabolism and circadian clock regulation in activated, highly proliferative cells. 

## 2. Metabolic Reprogramming in Cancer, Immune, and Endothelial Cells

The Warburg effect was first described in tumour cells by Otto Warburg (Warburg, 1925), who observed that malignant cells exhibited an elevated level of glycolytic activity even if a sufficient amount of oxygen was available. In the early 2000s, metabolic reprogramming from oxidative phosphorylation to the Warburg effect was demonstrated in immune cells, specifically naïve lymphocytes [6]. Later, these metabolic changes were observed in other immune cells especially within the adaptive immune system. Additionally, the important role of glycolytic metabolism was identified in highly proliferative cells, including pluripotent stem cells [7] and activated endothelial cells [8]. 

There are several mechanisms responsible for metabolic reprogramming toward glycolysis. Among them, hypoxia inducible factor-1α (HIF-1α) is the most highlighted factor [9], although several other factors are involved. It is well known that HIF-1α positively regulates the expression of glucose transporters and key glycolytic enzymes, thereby enhancing the glycolytic rate in cells. A major regulator of HIF activity is the oxygen level. Under normoxic conditions, the HIF-1α subunit is hydroxylated at proline residues by the prolyl hydroxylase domain-containing enzymes, leading to ubiquitin-mediated degradation in proteasomes. Oxygen, a substrate for the PHD enzyme, decreases HIF-1α hydroxylation in a hypoxia condition, resulting in an increased accumulation of HIF-1α. Subsequently, HIF-1α translocates to the nucleus and forms a transcriptional complex with p300 and, by binding to the hypoxia responsive element, affects the expression of multiple genes [9].

Additionally, HIF-1α is responsible for the suppression of mitochondrial function, leading to the Warburg phenotype. Firstly, HIF-1α increases the expression of enzyme pyruvate dehydrogenase kinase 1 (PDK1), leading to phosphorylation and the suppression of PDH activity [10]. Therefore, increased HIF-1α activity reduces acetyl-CoA levels and the subsequent tricarboxylic acid (TCA) cycle. Secondly, HIF-1α can affect the activity of miR-210, which negatively targets proteins associated with mitochondrial functions and suppresses their expression [11]. Moreover, HIF-1α is responsible for mitochondrial autophagy, resulting in a decreased number of mitochondria [10]. 

Glycolysis is closely related to the AMP-activated protein kinase (AMPK), which allows cell proliferation only under conditions of sufficient energy supply. Changes in the AMP/ATP ratio activate AMPK kinase, which induces phosphorylation of p53 that is required for AMPK-regulated cell cycle arrest. Mutations in p53 abolish the AMPK-mediated cell cycle arrest, leading to cell proliferation also in low glucose conditions [12]. Overall, p53 has a negative effect on the transcriptional activity of gene promoters for glucose transporters GLUT1 and GLUT4. Therefore, the mutation in *p53* has an opposite effect, resulting in increased glucose uptake and glycolysis [13]. 

Metabolic reprogramming can also be regulated by post-transcriptional modifications. Expression of miR-34a, which targets glycolytic enzymes such as hexokinase 1, hexokinase 2, and PDK1, can be suppressed by p53 in pancreatic ductal adenocarcinoma cells [14]. Additionally, it was confirmed that several metabolic enzymes, including glycolytic enzymes, can modulate the activity of chromatin-modifying enzymes, thus regulate the expression of genes and chromatin structure, as well as histone modification. Among them, fructose-1,6-bisphosphatase (FBP1), glyceraldehyde-3-phosphate dehydrogenase (GAPDH), PFKFB4 and pyruvate kinase M2 isoform (PKM2) play an important role. Pyruvate kinase M2 can regulate the gene expression by phosphorylation of histone H3 threonine 11 and activation of β-catenin and HIF-1. Enzyme PFKFB4 stabilizes the recruitment of steroid receptor co-activator protein 3 to the target gene promoters. Moreover, FBP1 can bind to HIF-1α and HIF-1β and negatively regulate the gene expression of HIF-1 targeted genes [15]. 

### 2.1. Cancer Cells 

Metabolic reprogramming in cancer cells was recognized as one of the hallmarks of cancer progression, which is responsible for metabolic changes including increased levels of glycolysis, glutaminolysis, amino acid, and lipid metabolism, and induction of the pentose phosphate pathway [16]. It is assumed that OXPHOS is not completely suppressed in tumour cells, as approximately 5% of pyruvate is further metabolized in this metabolic pathway. Decreased ATP production by aerobic glycolysis is compensated by increased levels of metabolism, while the glucose uptake requirements are met by an increased expression of glucose transporters, especially GLUT 1–4 [17]. 

Most of the glycolytic enzymes that regulate enhanced glycolysis are controlled by HIF-1α and the oncogenes MYC and RAS. In tumour cells, c-MYC and HIF-1α are the main inducers of glycolysis and promote the expression of key glycolytic enzymes, among them HK2, PFK1, and lactate dehydrogenase A (LDHA). Oncogene c-MYC regulates glycolytic gene expression in normoxia, and conversely, HIF-1α is functional in a hypoxic condition. This coordinated action allows cells to proliferate continuously under fluctuating oxygen levels [18]. 

In healthy cells, tumour suppressor p53 regulates glucose metabolism by direct inhibition of glucose transporter expression. However, in cancer cells, insufficient p53 regulation contributes to an increased expression of cancer-associated glycolytic genes. Additionally, loss of p53 function can lead to increased glycolysis by downregulation of TIGAR (TP53-induced glycolysis and apoptosis regulator). Under normal conditions, TIGAR slows down glycolysis by converting fructose-2,6-bisphosphate, an allosteric activator of PFK1 enzyme back to fructose-1-phosphate [19]. 

It was confirmed that expression of glucose transporters, especially GLUT3, is controlled by NF-κB, whose activation can be blocked by tumour suppressor p53. Therefore, reduced GLUT3 expression is followed by a decreased level of glycolysis. Mutation in *p53* enhances the expression of GLUT3 mediated by NF-κB pathways, thereby facilitating glycolysis [20]. 

### 2.2. Immune Cells 

Immune cells such as regulatory, memory, or effector cells undergo metabolic reprogramming to cover nutritional, metabolic, and biosynthesis requirements [21]. It is assumed that increased glycolysis provides carbon substrates for side metabolic pathways, such as the pentose phosphate pathway and serine biosynthesis pathway, necessary for nucleotide and lipid synthesis [22]. 

Depending on the activation in response to external signals, macrophages are divided into two subtypes: M1 macrophages activated in response to stimulation by interferon-γ (IFN-γ) and lipopolysaccharide and M2 macrophages activated in response to stimulation by interleukins (IL-4, IL-10, IL-13). Pro-inflammatory M1 macrophages are characterized by an increased level of glycolysis and a relatively low level of oxidative phosphorylation. Conversely, high fatty acid oxidation and OXPHOS are typical for M2 macrophages, which have metabolism comparable to unstimulated cells. M1 macrophages are characterized by the production of pro-inflammatory cytokines, thus promoting tissue injury, and show antitumor activity. On the contrary, M2 macrophages are characterized by anti-inflammatory properties resulting in tissue repair [23]. There are several mechanisms supporting metabolic reprogramming in M1 macrophages, including HIF-1α and target of rapamycin (mTOR) activation as well as downregulation of AMPK [6]. 

T cells are one of the first immune cells in which glycolytic reprogramming was described. T cells are able to switch from quiescent to a highly proliferative state depending on signals that induce the activation of intracellular signaling pathways [24]. Quiescent T cells are characterized by a high OXPHOS level required to maintain homeostatic processes. However, upon induction by growth factors, T cells switch to a proliferative active state and produce energy by aerobic glycolysis [6]. Glycolytic metabolism is induced by an antigen-specific signal and a co-stimulatory signal delivered by the CD28 receptor. T cell receptor (TCR) activation stimulates phosphatidylinol-3-kinase (PI3K) phosphorylation and subsequent activation of the serine-threonine kinase Akt. This kinase stimulates expression of a glucose transporter and its incorporation to the plasma membrane, thus facilitating glucose uptake to the cells [24]. Additionally, the increased rate of glycolysis is covered by the up-regulated activity of key enzymes, hexokinase, and phosphofructokinase [25]. Glycolysis inhibitors such as 2-deoxy-D-glucose (2-DG) inhibit the CD8 effector function that requires glycolytic reprogramming. The inflammatory and regulatory function of T cells is regulated by the balance between glycolysis and OXPHOS. T helper (T_H_) 1 and T_H_ 17 lymphocytes are dependent on aerobic glycolysis required for their differentiation and functions. On the other hand, regulatory T cells require enhanced glycolysis only in the initial steps of activation and proliferation and, subsequently, become glucose independent [6]. 

Naïve B lymphocytes in a quiescent state rely predominantly on OXPHOS for ATP production; however, upon activation, they increase lactate production and oxygen utilization because the up-regulated glycolysis is needed for antibody production and cell proliferation [6]. 

### 2.3. Endothelial Cells

Metabolic reprogramming toward glycolysis is also observed in endothelial cells, which can stay in a quiescent state for many years. Upon induction by growth factors such as vascular endothelial growth factor (VEGF), the endothelium starts proliferating and migrating and forming new vessels (*angiogenic switch*). Vessel sprouting during angiogenesis is a highly coordinated process that requires the migration of endothelial tip cells and the proliferation of endothelial stalk cells [4]. Despite the fact that endothelial cells are exposed to the circulating oxygen from the bloodstream, energy production is mainly dependent on glycolysis (around 85% of ATP). There are several reasons why endothelial cells rely predominantly on aerobic glycolysis in ATP production: (1) endothelial cells are not dependent on oxygen, so they can proliferate even in a hypoxia condition; (2) oxygen can diffuse to the surrounding tissues; (3) lower oxidative metabolism reduces the oxidative stress followed by decreased production of reactive oxygen species; (4) increased lactate production can promote angiogenesis [26]. 

The increased energy demand of endothelial cells is covered by the increased conversion of glucose to lactate. Glucose transport to endothelial cells is carried out by glucose transporters (GLUT-1, GLUT-3) or by sodium-glucose cotransport (SGLTs). Most of the glucose is transported to endothelial cells by GLUT1 [27]. After VEFG exposure, resting endothelial cells increase the level of glycolysis by increased expression of (1) GLUT1, (2) lactate dehydrogenase-A, which catalyses the conversion of pyruvate and NADH to L-lactate and NAD^+^, and (3) PFKFB3 [4]. 

Differentiation of endothelial cells into tip or stalk cells is dependent on VEGF-Notch signalling [28]. Tip cells are characterized by low Notch activity and high vascular endothelial growth factor receptor-2 (VEGFR-2) expression compared to stalk cells [29]. Glycolysis is the dominant metabolic pathway in migrating tip cells, and therefore, approximately 85% of ATP is produced by glucose oxidation. Stalk cells show increased Notch activity and decreased VEGFR-1 expression [30]. Glycolysis is also a necessary metabolic pathway for stalk cells with higher proliferative activity. Proliferating cells require increased production of macromolecules, which are produced in associated metabolic pathways [31]. 

Metabolic reprogramming in endothelial cells can be regulated by the HIF-1α factor, which induces increased expression of PDK1. Increased PDK1 expression results in the phosphorylation and subsequent inactivation of PDH, which blocks acetyl-CoA synthesis and, thus, suppresses the TCA cycle. Simultaneously, HIF-1α activates the VEGF signalling pathway involved in the regulation of glucose transporters and the expression of glycolytic enzymes [32]. In hypoxic pulmonary artery endothelial cells, HIF-2α downregulates the expression of *c-myc* and inhibits the expression of mitochondrial transcription factor A (TFAM), leading to reduced OXPHOS [33]. 

## 3. Physiology of the Circadian System 

Cellular metabolism, including glucose metabolism, is under strong circadian control. The mammalian circadian system is hierarchically organized with the central oscillator localized in the suprachiasmatic nucleus (SCN) of the hypothalamus that controls peripheral oscillators localized in nearly all cells of the body. Together, they govern 24 h rhythms of behaviour and physiology [34]. 

**Figure 1:**
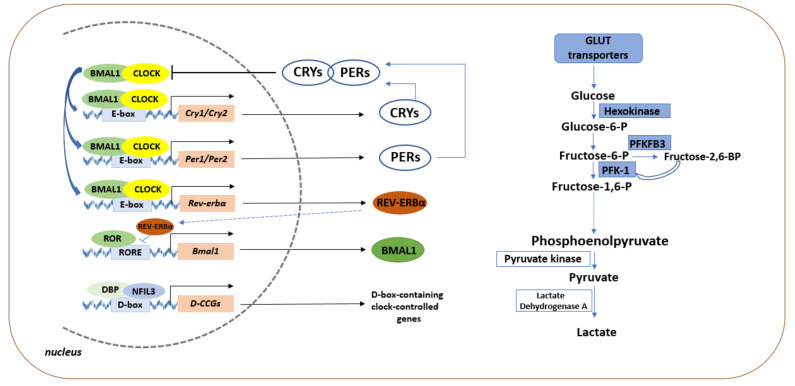
Schematic diagram of the transcription-translation feedback loop (TTFL) at the molecular level. First, the regulatory loop is mediated by the proteins CLOCK and BMAL1 that heterodimerize and bind to E-box of several clock genes, including *Per*, *Cry*, *Rev-erbα*, and other genes. The proteins PER and CRY form a complex that inhibits CLOCK and BMAL1 transcription. The activity of the second regulatory loop can be modulated by REV-ERBα, which induces and represses Bmal1 gene expression, respectively. In the third regulatory loop, the activator DBP induces expression of D-box containing clock-controlled genes. The DBP activator is inhibited by NFIL3, whose transcription is regulated by REV-ERBα and RORs. Regulatory loops ensure the rhythmic expression of the core clock genes, which can regulate cellular metabolism, including glycolysis. In this scheme, blue boxes indicate circadian-regulated metabolic genes that exhibit a circadian pattern. Abbreviations: BMAL1—brain and muscle ARNT-like 1; CLOCK—circadian locomotor output; Cry—cryptochrome; DBP—D-box binding protein; D-CCGs—D-box containing clock controlled genes; fructose-1,6-P—fructose 1,6-bisphosphate; fructose-6-P—fructose-6-phosphate; fructose-2,6-BP—fructose-2,6-bisphosphate; glucose-6-P—glucose-6-phosphate GLUT—glucose transporter; NFIL3—nuclear factor interleukin-3 regulated protein; Per—period; REV-ERBα- nuclear receptor subfamily 1 group D member 1; RORE—ROR responsive element; PFKFB3—6-phosphofructo-2-kinase/fructose-2,6-bisphosphatase; PFK-1—phosphofructokinase.

At the molecular level, circadian rhythms are generated in a cell-autonomous manner by the transcriptional translational feedback loop (TTFL) (Figure 1), which consists of clock genes whose protein products suppress transcription of other clock genes, resulting in both positive and negative feedback loops. Briefly, protein products of the core clock genes *Clock* (circadian locomotor output) and *Bmal1* (brain and muscle ARNT-like1), which represent a positive arm of the loop, heterodimerize and translocate to the nucleus. Here, they bind to the E-box promoter sequence of target core clock genes *Per1* and *Per 2* (Period) and *Cry1* and *Cry 2* (Cryptochrome) and initiate their transcription. PER and CRY proteins accumulate in the cytoplasm, form a complex, and translocate to the nucleus where it interacts with CLOCK/BMAL1 to inhibit its own transcription. The PER/CRY complex is eventually tagged for degradation via phosphorylation, releasing CLOCK/BMAL1 from suppression [35]. This general feedback control mechanism is specific in its duration because it takes approximately (circa) 24 h to complete. It can differ among different individuals, but it has a high repeatability in the same individual of a given age. 

There are several additional regulatory loops that stabilize the basic loop. Moreover, these additional pathways can predominantly determine the tissue specific circadian control of different organs or/and tissues [36]. One of the dominant supporting loops is formed by the nuclear receptor REV-ERBα, a member of the nuclear receptor superfamily (Subfamily 1 group D member 1, NR1D1) and a key circadian clock repressor that inhibits core clock activator *Bmal1* transcription 1 [37]. Importantly, *Rev-erbα* is highly expressed in metabolic tissues, with known functions in conferring circadian clock integration to glucose, lipoprotein, and bile acid metabolism [38]. REV-ERBα suppresses *Bmal1* transcription at ROR response elements (RORE) motifs (a nuclear orphan receptor related to the retinoic acid receptor), which is shared with retinoid acid receptor-related orphan receptor alpha (RORα). REV-ERBα represses, whereas RORα activates *Bmal1* gene transcription, and this antagonistic regulation elicits a *Bmal1* rhythmic oscillation. Moreover, *Rev-erbα* itself is a direct target of *Bmal1*, and the REV-ERBα-BMAL1 regulation constitutes a re-enforcing branch that enhances the robustness of the core clock machinery [37]. This loop may play a dominant role in the generation of circadian oscillations in some tissues, such as the liver and immune system, or during some pathophysiological conditions.

Important feedback loops are formed by transcription factors, so-called PAR proline and acidic amino acid-rich–basic leucine zipper proteins, such as DBP (D-box binding protein), HLF (hepatic leukemia factor), and TEF (thyreotropic embryonic factor). The gene encoding PER-1 contains, in the promoter region, a binding domain for DBP (D box) through which DBP stimulates its transcription. The E-box in the regulatory region of the *dbp*, in turn, mediates regulation of this gene by the BMAL1/CLOCK complex. The transcription factor E4BP4 (NFIL3) inhibits *per1* expression and oscillates in an opposite phase to DBP [39]. In this way, DBP can stimulate *per1* expression in one phase of a 24-h cycle, while E4BP4 inhibits DBP in the opposite phase, enhancing the stability of oscillations [40]. 

In addition to transcriptional regulation via E-box and D-box, circadian control of the metabolism is also mediated through interactions with tissue-specific metabolic transcription factors. Among them, a key role in metabolism control is played by peroxisome proliferator-activated receptor-γ coactivator (PGC1α), a transcriptional coactivator of nuclear receptor peroxisome proliferator-activated receptors (PPARγ), which is considered a master regulator of mitochondrial biogenesis and function [41]. Transcriptional coactivator PGC-1 alpha integrates the mammalian clock and energy metabolism [42], including OXPHOS. PGC1α and PGC1β stimulate the expression of mitochondrial genes, leading to increased fatty acid β-oxidation, Krebs cycle, and oxidative phosphorylation. Deficiency in PGC1α is followed by attenuated oxidative metabolism; therefore, in skeletal muscle, AMPK still remains activated, reflecting the energy deficit. However, it is still unclear whether a loss of PGC1α in mice leads to a metabolism switch toward glycolysis [43]. 

Equally important and less understood is the circadian control through post-transcriptional pathways, such as NAD^+^-dependent protein deacetylation [39], which plays the key role in determining a response to changing nutrient conditions. Indeed, abundant evidence demonstrates a strong connection between peripheral circadian clocks and basal metabolic processes, such as glucose metabolism [44]. However, the precise molecular mechanisms by which the circadian clock controls individual metabolic pathways and responds to nutrients still remain unclear.

## 4. Circadian Regulation of Glucose Metabolism 

The TTFL generates the circadian oscillations in a similar manner in the master circadian clock as in peripheral cells, including the heart, liver, pancreas, muscle, and white adipose tissue. Entrainment of peripheral clocks by the central circadian clock represents a mechanism by which peripheral tissue physiology can be entrained to central timing originating from the SCN [45]. The central oscillator SCN is entrained predominantly by the light/dark cycle, while the feeding/fasting cycle can synchronize peripheral clocks, especially in the liver, in which they control approximately 10% of the transcriptome and have an important role in the circadian alignment of metabolism [46]. Thus, altered timing of food intake in relation to the light:dark cycle can disrupt the synchrony between the brain and peripheral clocks [47] and lead to metabolic dysfunction, such as obesity, glucose intolerance, and cancer [48]. 

As mentioned above, circadian clocks contribute to the control of crucial metabolic pathways, including glycolysis; therefore, circadian rhythm disruption is associated with metabolic imbalance [49]. Glucose homeostasis is controlled by the hypothalamic clock localized in the SCN and peripheral clocks in the liver, muscle, pancreas, and white adipose tissue. Blood glucose is obtained mainly from the diet during the active phase and mainly from endogenous glucose production in the liver during the resting phase [50]. 

Glucose uptake shows a 24-h rhythm, with a peak at the beginning of the active phase and the lowest level at the beginning of the passive phase. In nocturnal rodents, the peaks occur in opposite phases than in diurnal humans. Additionally, the 24-h rhythm of glucose uptake corresponds with the glucose concentration in plasma. After the lesion of SCN, the rhythm in glucose uptake and glucose concentration was diminished, and additionally, the rhythm in insulin-dependent tissue sensitivity was eliminated [51]. Therefore, chronodisrupted patients show disruption of rhythms in plasma glucose and insulin levels. Moreover, genetic analyses showed an association between CRY and PER2 and the glucose concentration in the blood [52]. 

Glucose uptake into the cell depends on the expression of glucose transporters and their trafficking on the cell surface. Corpe and Buran [53] demonstrated that glucose transporter mRNA, among them GLUT-5, GLUT-2, and SGLT-1, exhibits a 24-h rhythm, with the peak occurring before the peak of the feeding rhythm. Expression of intestinal transporters varies on a daily basis, and the peak in SGLT1 activity corresponds with the peak level of the SGLT1 protein. Temporal changes in the *Sglt1* mRNA level persisted with a lower amplitude and peak in the ileum in comparison to jejunum [54]. Mice with a muscle-specific clock disruption exhibit impaired glucose uptake, which is probably mediated by the insulin-dependent GLUT4 transporter because its protein level was reduced in this model. Additionally, the level of OXPHOS was reduced and glycolytic intermediates were metabolized inside metabolic pathways because of the decreased activity of pyruvate dehydrogenase [52]. 

The circadian clock located in hepatocytes plays an important role in the regulation of glucose homeostasis by control of glucose turnover and gluconeogenesis. The level of hepatic glycogen shows daily variations in humans depending on the activity of circadian regulated enzymes [55]. Two enzymes, glycogen synthase and glycogen phosphorylase, are rate-limiting enzymes that control the process of glycogenesis and glycogenolysis. In nocturnal rats, glycogen synthase 2 (GYS2) shows a circadian rhythm with a peak late at night. The activity of GYS2 is modulated by the hormonal signals, including insulin and glucagon as well as glucocorticoids [56]. Circadian expression of *Gys2* is regulated through E-box by the transcription factor CLOCK. 

Moreover, the activity of glycolytic enzymes shows a circadian oscillation. The expression of the key glycolytic enzyme PFKFB3 at the mRNA and protein level in tongue cancer cells shows circadian oscillations controlled by the transcription factor CLOCK. The peak mRNA levels were achieved in the early light phase between ZT5 and ZT9, and lower levels were achieved between ZT17 and ZT21 [57]. The study with tongue cancer cells confirms that PFKFB3 inhibition at its peak levels significantly decreases cell proliferation and lactate production. Results offer a new insight into the chronopharmacological approach of cancer treatment. Cancer chronotherapy, e.g., administration of anticancer drugs at certain times of the day to reach maximum efficacy and minimum side effects, has been an attractive possibility for long time [58], but large multicenter trials have not produced beneficial outcomes. Therefore, future mechanism-based studies are needed to provide information necessary for devising rational chronochemotherapy regimens [59]. Since growing solid tumours contain different types of cells and are abundantly vascularized, the anticancer chronotherapy targeted on inhibition of glycolysis in proliferating cancer and endothelial cells can represent a useful strategy. Our recent study confirms that the possibility to target metabolic pathways at a specific time may be a promising approach. We found that administration of glycolysis inhibitor 1-(4-pyridinyl)-3-(2-quinolinyl)-2-propen-1-one (PFK15) at different times can result in a significant reduction in the tumour progression [60]. Another study on nude mice with implanted breast tumour cells into the femoral artery proved an increased arterial glucose uptake by tumour cells and lactate concentration in the blood. Increased lactate production reflects a higher level of glycolysis during the passive phase, with peak levels two hours before the lights are off. Additionally, based on the [3H] thymidine incorporation assay, the cell proliferation and number of tumour cells were confirmed to increase during the light phase [61]. Different effects observed after glycolysis inhibition may be related to circadian processes with the peak during the passive phase. 

Aerobic glycolysis is considered a main metabolic pathway in brain astrocytes. In human astrocytes, increased expression of CLOCK and BMAL1 is linked with suppression of the protein level of HK1 and LDHA. Moreover, increased BMAL1 expression suppresses the extracellular acidification rate (ECAR) because of attenuated lactate production. Additionally, CLOCK and BMAL1 activation is responsible for caspase-3 mediated apoptosis in human astrocytes. A recent study suggests that reduced glucose metabolism in astrocytes may be associated with Alzheimer’s disease and cognitive dysfunction [49]. 

The oncoprotein c-MYC is a transcriptional activator involved in the control of cell cycle progression and tumorigenesis and is controlled by the circadian clock [62]. Several studies suggest that clocks can control *c-Myc* transcription and MYC protein stability. Cryptochromes, as the components of the negative arm of the TTFL were reported to bind to phosphorylated c-MYC, target it to ubiquitylation by FBXL3 and degradation by the proteasome [63]. However, in Cry 1/2 knockout mice, *c-Myc* transcription and protein levels were depressed and were elevated in BMAL1 knockouts [64]. These authors suggest that *c-Myc* is a second-order clock-controlled gene regulated through β-catenin, which binds the T-cell factor/lymphoid enhancer factor (TCF/LEF) family of transcription factors resulting in high expression of c-Myc. Further studies are needed to elucidate if the genetic background can affect *c-Myc* expression and explain the contradictory results. Overexpression of *c-Myc* results in up-regulated expression of several genes involved in glycolysis, glutaminolysis, and oxidative phosphorylation. In vehicle-treated MYC-OFF U2OS cells, the level of HK1 and HK2 oscillated in phase with glucose consumption. Conversely, in MYC-ON U2OS cells showing the increased activity of MYC, HK2 was induced and did not oscillate. The observed changes are in line with the decrease in MYC-induced BMAL1 and the increase in the PER2 protein level [65].

### 4.1. Transcription Factors CLOCK and BMAL1

Recently, it was confirmed [66] that the transcription factors CLOCK and BMAL can affect glioma cell proliferation, migration, and metabolic reprogramming. Loss of CLOCK and BMAL affected the glioblastoma cell cycle and apoptosis followed by decreased cell proliferation. In embryonic fibroblasts isolated from mice (MEF) lacking the transcriptional activators CLOCK and BMAL1, decreased FAO and NAD^+^ concentrations were observed, and conversely, MEF deficient in repressor CRY1 and CRY2 showed increased FAO and NAD^+^ concentrations. Additionally, *Bmal1^−/−^* MEF fibroblasts cultivated in glucose-containing medium released more lactate, indicating a cell dependence on glycolysis. Moreover, the expression of key glycolytic enzymes, including PFK1, increased [67]. 

Interestingly, in embryonic fibroblasts isolated from *Bmal1*^−/−^ mice, an elevated glycolysis level and increased lactate production was observed, similar to the metabolic phenotype observed in cancer cells. This metabolic change to the glycolytic phenotype is regulated through the clock-mediated transcriptional regulation of genes encoding glycolytic enzymes [67]. Additionally, the reduced concentration of NAD^+^ was observed in embryonic fibroblasts due to the downregulated transcription of nicotinamide phosphoribosyltransferase (NAMPT), an enzyme catalysing the first step in the biosynthesis of NAD. The increased level of NAD is associated with an inflammatory state, including cancer and age-related diseases [68]. Lack of NAD^+^ supports the shift from OXPHOS to aerobic glycolysis and attenuates lipid oxidation in mitochondria [69]. Moreover, the activity of sirtuins is dependent on NAD^+^ concentrations. Reduction of the sirtuin 3 (SIRT3) concentration leads to hyperacetylation of mitochondrial enzymes followed by attenuation of oxidative phosphorylation [69]. 

Key immune system parameters (e.g., cells, hormones, and cytokines) circulating in the blood are under circadian control, exhibit circadian rhythms, and oscillate according to the day-night changes. The metabolism of immune cells can change dynamically according to the presence of cytokines, pathogen molecules, and metabolites [70]. It is assumed that pyruvate kinase M2 (PKM2) is a transcriptional target of BMAL1 in macrophages; therefore, loss of BMAL1 induces PKM2 expression followed by increased lactate production. Upregulated lactate production due to PKM2-mediated lactate metabolism contributes to the regulation of an inflammatory response and the development of sepsis. In double knockout mice (*Bmal1^Mye−/−^*; *Pkm2^Mye−/−^* mice), the loss of *Pkm2* decreased the lactate level and increased cell survival due to the reduced glycolysis and increased OXPHOS. PKM2 knockout did not affect the expression of *Bmal1*, *Per1*, and *Per2* mRNA in macrophages isolated from *Bmal1^Mye−/−^*; *Pkm2^Mye−/−^* mice [71]. Transition of PKM2-dependent aerobic glycolysis to OXPHOS may improve the course of the sepsis. 

Circadian clock disruption is related to different disorders, including inflammatory and metabolic diseases. Deng and colleagues [71] confirmed a direct interaction between the circadian system, cell metabolism, and immune system in the development of sepsis. As myeloid cells, e.g., monocytes and macrophages, are a key component of the innate immune response, specific mice with BMAL1 knockout in myeloid cells (*Bmal1^Mye−/−^* mice) were generated. Loss of BMAL1 in myeloid cells negatively affected the circadian oscillators in macrophages; however, the BMAL1 protein level in other tissues was unchanged, proving the cell specific knockout in myeloid cells [71]. Additionally, BMAL1 knockout in mice leads to B cell maturation defects and a reduced number of B cells in the circulating blood [72].

### 4.2. REV-ERBα

Another clock-transcription factor regulating cell metabolism and proliferation is REV-ERB, which participates in glucose and lipid metabolism [73]. REV-ERBα is expressed in tissues such as the liver, adipose tissue, pancreas, and muscle, where it modulates glucose, lipid, and bile acid metabolism, adipogenesis, and the inflammatory response. In mouse hepatocytes and human hepatoma cells, REV-ERBα regulates glucose metabolism through the activity modulation of enzyme glucose-6-posphatase. Moreover, REV-ERBα activation attenuates the level of plasma and cellular glucose [74]. 

In cancer cell lines, REV-ERBα up-regulation is associated with protooncogene MYC followed by reduced BMAL1 levels and loss of circadian control of glucose metabolism [65]. In glucose metabolism, REV-ERBα inhibits the expression of the rate-limiting enzymes HKII and PFKFB3, thus reducing cell proliferation. REV-ERBα is recruited to the promotor of the human *pfkfb3* gene and suppresses its expression. However, this effect was not observed in the promotor of the *hkII* gene. Gene expression of *pfkfb3* and *hkII* modulated by REV-ERBα is BMAL1 independent but depends on its DNA binding domain (DBD) [75]. Additionally, the use of SR8278, a synthetic REV-ERBα antagonist, reduces the level of glycolysis and raises the intracellular level of lactate [74]. 

### 4.3. Transcription Factor HIF

The circadian system reacts not only to the light-dark cycle but also to the rhythmic feeding [47] and oxygen level. Recent studies show that the circadian clock interacts with the transcription factors HIF1α and HIF2α, which are known as oxygen-sensing transcription factors [76]. They are induced by hypoxic conditions and activate the transcription of multiple genes involved in angiogenesis, such as VEGF, hormone erythropoietin stimulating red blood cell formation, glucose transporters (GLUT 1 and GLUT 4), and glycolytic enzymes, such as LDHA [77], pyruvate dehydrogenase [78], and glyceraldehyde-3-phosphate dehydrogenase [79]. This network is important in several physiological and pathological tumorigenesis conditions. The HIF regulated response is present, for example, in skeletal muscle, in which it activates glucose uptake and lactate production in response to exercise [80]. In cancer tissue, the activated HIF pathway drives angiogenesis in the growing tumour [81]. Numerous studies with knock-out mice demonstrate the interaction between the circadian clock and the HIF pathway [67]. It is expected that BMAL1 and HIFα can directly interact and form a heterodimer, which directly controls the transcriptions of some genes in vitro [82]. However, an indirect mechanism is also plausible because the circadian clock participates in the control of glycolysis, Krebs cycle, fatty acid oxidation, and election transport [83]. Thus, it is possible that the circadian clock is also able to control HIFα activity and stability via control of the metabolism or important signalling metabolites [84]. On the contrary, the relationship between HIF and TTFL can be reciprocal because, in several experimental models, hypoxia can reduce the amplitude of circadian rhythms [85,86] or influence adaptations to circadian phase shifts [87]. 

**Figure 2:**
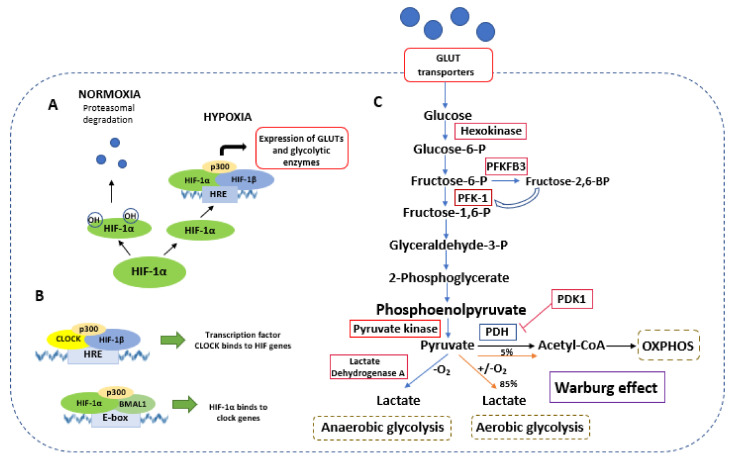
Crosstalk between HIF-1, circadian clocks, and glycolysis. (**A**) Under a normoxic condition, HIF-1α is ubiquitinated and degraded. In a hypoxia condition, HIF-1α translocates to the nucleus, binds to the HIF-1β and p300, and forms the HIF complex. This complex binds to hypoxia-response elements (HRE) and regulates the expression of glucose transporters and glycolytic enzymes. (**B**) HIF can interact with circadian pathways. CLOCK can interact with HIF-1β and p300 and binds to HRE. Additionally, HIF-1α can colocalize with BMAL1 to increase the expression of HIF- and clock-controlled genes. (**C**) Schematic representation of glycolytic metabolism regulated by the HIF-1α factor. Red boxes indicate gene expression regulated by HIF-1α. Pyruvate can be metabolized into lactate in a hypoxic condition (anaerobic glycolysis) or a condition of sufficient oxygen (aerobic glycolysis). In the process of OXPHOS, pyruvate is metabolized to Acetyl-CoA. Abbreviations: BMAL1—brain and muscle ARNT-like 1; CLOCK—circadian locomotor output; GLUT—glucose transporter; glyceraldehyde-3-P—glyceraldehyde-3-phosphate; HRE—hormone responsive element; HIF—hypoxia inducible factor; PFKFB3—6-phosphofructo-2-kinase/fructose-2,6-bisphosphatase; PFK-1—phosphofructokinase; fructose-1,6-P—fructose 1,6-bisphosphate; fructose-6-P—fructose-6-phosphate; fructose-2,6-BP—fructose-2,6-bisphosphate; glucose-6-P—glucose-6-phosphate; OXPHOS—oxidative phosphorylation; PDH—pyruvate dehydrogenase; PDK1—pyruvate dehydrogenase kinase 1.

HIF-1α and HIF-1β as well as BMAL1 and CLOCK are members of the transcription factor family bHLH-PAS, sharing structural similarities. These factors can respond to physiological as well as environmental signals. Interactions between circadian clocks and hypoxia were identified in previous works [76] (Figure 2). It was confirmed that HIF-1 can contribute to the expression of *per2* and *cry1* genes. As a heterodimer, HIF-1α and BMAL1 can bind to the E-box and increase expression of HIF-1 target genes. HIF-1α can bind directly to the E-box of some circadian genes. In human bone osteosarcoma epithelial cells (U2OS), interaction between HIF-1α and BMAL1 increases the expression of *per2* except for the HIF-1 target genes. In turn, the transcription factors CLOCK and BMAL1 may promote the expression of the HIF-1α gene [82]. 

Under a normoxic condition, increased expression of genes related with anaerobic glycolysis and lactate production was documented in the *Bmal1^−/−^* liver. Interestingly, in *Bmal1^−/−^* myotubes, lactate production and HIF-1α gene expression were reduced. In myotubes, an extracellular medium acidification rate (ECAR) showed circadian rhythmicity with an opposing phase to the rhythms of fatty acid oxidation. This observation indicates that glycolytic and oxidative metabolism are clock-controlled in skeletal muscle [88]. 

M1 macrophage activation induces BMAL1 mRNA and protein expression, with a peak 12 h after stimulation. BMAL1 may affect the activation of macrophages through direct interaction with HIF-1α. Both BMAL1 and HIF-1α regulate different metabolic pathways, while HIF-1α predominantly regulates aerobic glycolysis and BMAL1 regulates metabolism toward oxidative phosphorylation [70]. In macrophages, mitochondrial production of reactive oxygen species can be enhanced by succinate dehydrogenase after loss of BMAL1 function, followed by HIF-1α stabilization, which contributes to tissue inflammatory damage [89]. Additionally, loss of BMAL1 function induces higher expression of genes encoding plasma membrane transporters for amino acids and enzymes involved in the breakdown of amino acids, leading to metabolic reprogramming toward catabolism of amino acids. On the other hand, HIF-1α induces expression of enzyme LDHA and arginase 1 (Arg1) [70].

### 4.4. Melatonin

There are several lines of evidence that metabolic changes toward the glycolytic phenotype can be influenced by circadian melatonin production. Melatonin can contribute, through the inhibition of HIF-1α, to the down-regulation of pyruvate dehydrogenase kinase, which inhibits the pyruvate dehydrogenase complex catalysing the conversion of pyruvate to acetyl-CoA. Therefore, pyruvate is metabolized in the cell cytoplasm to lactate, and other metabolic pathways, such as the pentose phosphate pathway, are upregulated. Additionally, melatonin contributes to the conversion of proinflammatory M1 macrophages to anti-inflammatory M2 macrophages followed by changes in the glucose metabolism (from aerobic glycolysis to oxidative phosphorylation) [90]. This indicates that melatonin can also modulate metabolism in non-cancer cells.

During the daytime, xenografted human mammary cancer cells in rats showed the Warburg phenotype characterized by an increased glucose uptake and glucose metabolism. However, at night, these cells preferred oxidative phosphorylation. The day-night difference was not observed in cultured cancer cells, which were not exposed to the rhythmic melatonin condition. It is assumed that changes in melatonin levels in rats exposed to light at night were responsible for the metabolic alterations observed in these cells. Mitochondrial oxidative phosphorylation did not occur, and glucose metabolism was also observed during the nighttime. After changes in the melatonin level, cells display aerobic glycolysis during the day and night [61].

## 5. Circadian Rhythms in Control of Oxidative Phosphorylation 

Mitochondria play a central role in the process of oxidative phosphorylation. Except for oxidative phosphorylation, mitochondria play an important role in lipid biosynthesis and calcium homeostasis, processes which may be under circadian clock control. In rat hepatocytes, the volume and shape of the mitochondria can oscillate under light and dark conditions. Additionally, some genes included in mitochondrial dynamics are expressed in a circadian manner, and their expression is controlled by BMAL1. Hepatocytes isolated from mice harvested at different times during the day exhibited higher levels of respiration during the dark in comparison to the light phase in the presence of pyruvate. However, in hepatocytes isolated from the liver of BMAL1 knockout mice, this effect was not observed [91], showing a key role of circadian clocks in the control of energy metabolism. 

Additionally, mitochondrial genes encoding subunits of complex I, IV, and V also show transcriptional circadian oscillations. The concentration of mitochondrial enzymes changes during the daytime in a circadian manner, indicating a potential post-transcriptional mechanism [92]. 

Another study [92] confirmed circadian control of oxidative phosphorylation was dependent on dynamin-related protein 1 (DRP1). The activity of DRP1 is regulated via phosphorylation at serine residue 637 (Ser637) and subsequent activation/inactivation. DRP1 phosphorylation at Ser637 shows 24-h rhythms with the peak at CT12, the beginning of the subjective night. On the contrary, the level of total DRP1 protein did not show circadian oscillation in cells and lysates from brains of mice kept in constant darkness. 

## 6. Conclusions

Glycolysis is the main metabolic pathway for energy production in highly proliferative cells. This metabolic switch from oxidative phosphorylation to glycolysis was, for the first time, described in cancer cells and later in activated immune and endothelial cells. Metabolic reprogramming is a promising target for the treatment of different diseases, from cancer progression to autoimmune diseases. Inhibition of glucose metabolism is possible at several steps, among them the enzyme PFKFB3 is one of the promising targets. Therefore, several compounds have been developed to block its activity. Since this enzyme and other steps of glucose metabolism are under circadian control, the circadian approach to metabolic reprograming is considered in this review. Circadian rhythms are generated by the redundant transcriptional translational feedback loops, and several clock and clock-controlled genes regulate glucose metabolism. Therefore, a chronopharmacological approach can be promising in the control of metabolic reprogramming and the treatment of several diseases related to glycolysis, including cancer.

## Data Availability

Not applicable.
